# Prenatal and Postpartum Evening Salivary Cortisol Levels in Association with Peripartum Depressive Symptoms

**DOI:** 10.1371/journal.pone.0135471

**Published:** 2015-08-31

**Authors:** Stavros I. Iliadis, Erika Comasco, Sara Sylvén, Charlotte Hellgren, Inger Sundström Poromaa, Alkistis Skalkidou

**Affiliations:** 1 Dept. of Women's and Children's Health, Uppsala University, 751 85, Uppsala, Sweden; 2 Dept. of Neuroscience, Uppsala University, 751 24, Uppsala, Sweden; University of Rennes-1, FRANCE

## Abstract

**Background:**

The biology of peripartum depression remains unclear, with altered stress and the Hypothalamus-Pituitary-Adrenal axis response having been implicated in its pathophysiology.

**Methods:**

The current study was undertaken as a part of the BASIC project (Biology, Affect, Stress, Imaging, Cognition), a population-based longitudinal study of psychological wellbeing during pregnancy and the postpartum period in Uppsala County, Sweden, in order to assess the association between evening salivary cortisol levels and depressive symptoms in the peripartum period. Three hundred and sixty-five pregnant women from the BASIC cohort were recruited at pregnancy week 18 and instructed to complete a Swedish validated version of the Edinburgh Postnatal Depression Scale at the 36th week of pregnancy as well as the sixth week after delivery. At both times, they were also asked to provide evening salivary samples for cortisol analysis. A comprehensive review of the relevant literature is also provided.

**Results:**

Women with postpartum EPDS score ≥ 10 had higher salivary evening cortisol at six weeks postpartum compared to healthy controls (median cortisol 1.19 vs 0.89 nmol/L). A logistic regression model showed a positive association between cortisol levels and depressive symptoms postpartum (OR = 4.1; 95% CI 1.7–9.7). This association remained significant even after controlling for history of depression, use of tobacco, partner support, breastfeeding, stressful life events, and sleep problems, as possible confounders (aOR = 4.5; 95% CI 1.5–14.1). Additionally, women with postpartum depressive symptoms had higher postpartum cortisol levels compared to both women with depressive symptoms antenatally and controls (*p* = 0.019 and *p* = 0.004, respectively).

**Conclusions:**

Women with depressive symptoms postpartum had higher postpartum cortisol levels, indicating an altered response of the HPA-axis in postpartum depression.

## Introduction

### Peripartum depression

Peripartum depression is a disorder encompassing depressive episodes occurring during pregnancy and the perinatal period. According to the fifth edition of the Diagnostic and Statistical Manual of Mental Disorders (DSM-5), peripartum depression refers to a depressive episode with onset during pregnancy or within the first four weeks following delivery [1]. Despite this restriction, in clinical settings, a diagnosis of postpartum depression (PPD) often refers to a major depressive episode with onset within the first 12 months after delivery. The prevalence of PPD is reported to be between 10% and 15% according to most studies [2, 3]. Rates vary widely between studies and can be partly attributed to the different criteria used to define perinatal and postpartum depression. Risk factors for developing PPD include a history of previous perinatal or major depressive episode [4, 5], stressful life events in the previous 12 months, lack of partner support during pregnancy, obstetric complications, young age, unplanned pregnancy and low socioeconomic status [3, 6–8]. However, the biological mechanisms underlying peripartum depression have not yet been clearly elucidated [9].

### Hormonal aspects

To date, several studies have focused on endocrine factors associated with the pathophysiology of peripartum depression, including Hypothalamus-Pituitary-Adrenal (HPA)-axis hormones [10, 11]. Normal pregnancy itself is associated with profound hormonal fluctuations. In fact, during an uncomplicated pregnancy, mean levels of baseline salivary cortisol start to rise gradually, soon after conception and during late pregnancy surpass those in non-pregnant women by more than two times [12, 13]. This HPA-axis hyperactivity is mostly a result of the increased levels of circulating Corticotropin Releasing Hormone of placental origin (pCRH) as well as the concurrently decreasing levels of corticotropin releasing hormone binding protein during late pregnancy. These physiological changes contribute to elevated levels of bioactive free Corticotropin Releasing Hormone (CRH) and consequential hyper secretion of Adrenocorticotropic Hormone (ACTH) and cortisol [14]. Meanwhile, hypothalamic CRH is down regulated, leading to low cortisol levels after partus and placental expulsion [10, 14, 15]. While in most cases salivary cortisol concentration is normalised within a two-week period after delivery, an abnormal adjustment to this state may result in a persistent HPA-axis suppression and hypocortisolemia, which is believed to increase the susceptibility to PPD [16]. Additionally, it has been pointed out that perinatal depression is a heterogeneous disorder with substantial differences in the etiology and clinical expression of depressive symptoms occurring before and after delivery and has been associated with both hypo- and hypersensitivity of the HPA axis [16, 17].

### Literature review

To date, around 40 studies have investigated cortisol levels in women during the peripartum period in relation to mood disorders (Table 1). However, studies are characterized by high heterogeneity. Indeed, sample size ranged largely between studies (average sample size, n ≈ 86), with the largest including 353 women [18], and the smallest 17 [10]. Longitudinal hormone measurements were employed by the majority of studies [10, 12, 19–39]. Most of the studies have chosen to assess salivary cortisol [12, 19, 20, 27, 30, 32, 33, 35–37, 40–47] and/or blood cortisol [10, 12, 21–24, 26, 27, 29, 31, 34, 35, 37–39, 42, 45, 48–53], while in a minority of studies, study subjects have been sampled for urinary cortisol analyses [18, 25, 31, 54]. Differences also exist regarding the time of assessment. Most of the studies have measured cortisol levels during the third trimester of pregnancy [12, 18–22, 24, 25, 27–29, 31, 34–37, 39, 41, 43, 44, 47, 49, 51–53] and up to 12 weeks postpartum [10, 12, 21, 23, 24, 27–35, 39, 40, 42, 45, 48, 50, 53]. Furthermore, variations exist in the time point during the day when hormonal assessment was carried out as most studies have assessed morning samples or cortisol awakening response (CAR) [12, 18–21, 25–37, 39–46, 48, 50, 51, 53, 54], while others examined afternoon [12, 20, 21, 23, 24, 27, 32, 33, 37, 47, 49, 53] or evening cortisol [10, 12, 20, 27, 33, 38, 40, 45, 46, 52].

**Table 1:** Summary of studies assessing the relationship between peripartum cortisol levels and depression.

Study	Sample (*N*)	Age (years)	Hormonal assessment time	Mood Measure Method & Time	Groups	Modality/technique	Findings
**Glynn and Sandman, 2014[49]**	170	29.5	gw14-16 + 18–20 + 24–26 + 30–32 + ≥36	CES-D (gw14-16 + 18–20 + 24–26 + 30–32 + ≥36), EPDS (ppm3 + 6)	136 HC, 34 DEP	Afternoon plasma cortisol. Competitive binding solid-phase enzyme-linked immunosorbent assay	n.s.
**Hellgren *et al*., 2013 [19]**	134	30.94	gw35-39	MINI (gw35-39), EPDS (gw17 + 32 + 35–39)	57 HC, 39 DEPh, 38 DEP	Waking, 15min, 30min, 45min post waking. Salivary cortisol, cobas elecsys kit	n.s.
**Saleh el *et al*., 2013 [50]**	120	28.5	ppw1	EPDS (ppw1, ppm1 + 3 + 12)	60 HC, 60 PPD	Morning blood cortisol	PPD: greater drop in pp cortisol. Positive correlation between severity of PPD and cortisol
**Peer *et al*., 2013 [46]**	57	30	gw19-21	PRIME-MD PHQ, EPDS (gw19)	65 HC, 13 DEP	2 consecutive days: waking, 30min and 60min post-waking, 2100 h, 2200 h. Salivary cortisol, Salimetrics enzyme immunoassay kit	PND: higher evening cortisol
**Voegtline *et al*., 2013 [47]**	112	31.2	gw24-26 + 27–29 + 30–32 + 33–35 + 36–38	CES-D (gw24-26 + 27–29 + 30–32 + 33–35 + 36–38)		Mid-afternoon salivary cortisol. Enzyme Immunoassay	DEP gw30-32: higher cortisol
**Meliska *et al*., 2013 [52]**	29	25.6	≤gw34	SCID, HRS-D, EPDS, SIGH-SAD, BDI (≤gw34)	12 HC, 17 DEP	Evening plasma cortisol. Solid-phase Radioimmunoassay	Positive correlation between cortisol mesor (but not evening cortisol) and HRS-D score antenatally
**Salacz *et al*., 2012 [51]**	79	37.1	gw36-38	BDI (gw36-38)		Morning blood cortisol (1 hour after awakening). Radioimmunoassay	n.s.
**Giesbrecht *et al*., 2012 [20]**	83	32.42	gw6-37 x 3 days	POMS, EPDS, SCL-90-R (gw6-37)		Salivary cortisol, waking + 30min + 45min after + semi-randomly at 1100 h + 1600 h + 2000 h. Immunoassay	Within-person positive association between negative mood and cortisol
**O'Keane *et al*., 2011b [37]**	65	32.8	gw25 + 26 + gw36	SCID (inclusion), HAM-D, EPDS, BDI, BPRS (gw25 + 36)	38 HC, 27 DEP	Blood cortisol (gw25 +36, between 1100h and 1500 h), chemiluminescence immunoassay salivary cortisol (gw26, morning and evening x 3 days)	PND: positive correlation with cortisol
**O'Keane *et al*., 2011a [21]**	70	33.1	gw 36 + ppd1-6	SCID + EPDS + Blues Questionnaire		Blood cortisol (between 1100 and 1500h) Chemiluminescence immunoassay salivary cortisol	n.s.
**Meinlschmidt *et al*., 2010 [35]**	22	30.1	gw36 + ppw6	Mood questionnaire + STAI		Waking, 30min, 45min, 60min salivary cortisol with immunoassay, plasma cortisol with chemiluminescence assay	Salivary cortisol during pregnancy negatively correlated with ACTH and plasma cortisol and salivary cortisol responses to acute stressor ppw8
**Pluess *et al*., 2010 [36]**	53	30.7	gw15 + gw35/36	EPDS		Waking, 30min, 45min, 60min salivary cortisol. Luminescence immunoassay	n.s.
**Yim *et al*., 2009 [22]**	100	31.2	gw15 + 19 + 25 + 31 + 37	CES-D (gw19 + 25 + 31 + 37)EPDS (ppw8.7)	84 HC, 16 DEP	Blood cortisol. Radioimmunoassay	n.s.
**Taylor *et al*., 2009 [40]**	72	32.6 (HC) 24.7 (non pregnant HC), 32.5 (DEP)	ppw7.5	EPDS (ppw4)	30 HC, 21 non pregnant HC, 21 DEP	Waking, 30min, 3h, 12h post-waking salivary cortisol. Enzyme immunoassay	EPDS > 12: higher waking cortisol than HC and no cortisol rise at +30min compared to EPDS ≤ 12
**Evans *et al*., 2008 [41]**	180	27.4 (HC), 23.6 (DEP), 24.7 (ANX), 23.3 (DEP + ANX)	gw33-39	SCID (gm4-6)	121 HC, 16 DEP, 34 ANX, 9 DEP + ANX	Mid-morning salivary cortisol Radioimmunoassay	DEP: n.s.
**Field *et al*., 2008 [54]**	102	27.2	gw16-22	SCID, STAI, CES-D (gw16-22)	48 dysthymic, 54 MDD	Morning urinary cortisol Radioimmunoassay	Dysthymia: higher cortisol (gw18) than HC
**Groer and Morgan, 2007 [42]**	194	25.9 (HC) 23.6 (DEP)	ppw4-6	POMS-D (ppw4-6)	169 HC, 25 DEP	Early morning salivary and blood cortisol ELISA	DEP: lower salivary cortisol
**Jolley *et al*., 2007 [23]**	22	N/A	ppw6 + 12	PDSS	13 HC, 9 DEP	Afternoon serum cortisol. Antibody immunoassay	n.s.
**Shea *et al*., 2007 [43]**	66	30.5	gw25-33	EPDS or MADRS + STAI (gw25-33)	33 HC, 14 DEP	Waking, 30min, 60min salivary cortisol Enzyme immunoassay	n.s.
**Lommatzsch *et al*., 2006 [24]**	40	28	gw30 + 37, ppw1 + 8	EPDS (gw30 + 37, ppw1 + 8)	40 HC (pregnant)	Afternoon serum cortisol Electrochemiluminescence immunoassay	EPDS > 9: higher cortisol
**Field *et al*., 2006 [18]**	353	26.7	gw20 + 32	SCID, CES-D, STAI (gw20 + 32)	258 HC, 172 DEP	Morning urinary cortisol	DEP and ANX: higher cortisol
**Diego *et al*., 2006 [25]**	98	26	gw16-29	CES-D, STAI (gw16 + 29)		Morning urinary cortisol Radioimmunoassay	PND and ANX: positively correlated with cortisol
**Nierop *et al*., 2006 [44]**	57	21–35	13-31gw	EPDS (ppd2-27)	41 HC, 14 DEP	Morning salivary cortisol Chemiluminescence immunoassay	n.s. After stress: EPDS > 9 higher cortisol than EPDS ≤ 9
**Parry *et al*., 2003 [38]**	20	27	N/A	HRS-D, SCID, BDI, EPDS, visual analogue scales for mood	13 DEP (pp), 2 HC (pp), 3 DEP (preg), 2 HC (preg)	Evening plasma cortisol (between 1800 h and 2300 h)	PND & PPD: lower mesor cortisol
**Susman *et al*., 1999 [26]**	59	17.28	gw9-21	DISC (gw9-21 + gw32-34 + pw4-5)		Morning plasma. Radioimmunoassay	n.s.
**Magiakou *et al*., 1996 [10]**	17	32	ppw3 + 6 + 12	HRS-D (ppd2 + ppw2 + 3 + 6 + 8 + 12 + 16 + 20)	7 blues, 1 DEP, 9 HC	Plasma cortisol 1 min before and 5, 15, 30, 60, 90, 120 min after ovine CRH (2000 h) administration. Radioimmunoassay	n.s.
**Harris *et al*., 1996 [27]**	120	26.4	2w before delivery and ppw2 –ppd35	Stein Questionnaire for Blues (ppd0-10), EPDS, MADRS, Raskin scale, BDI (ppd35- 40)		gwx morning + evening; ppd1-5 morning + mid-day + evening; pp morning + evening salivary cortisol; ppd1 + 5 + 35 blood cortisol	PPD: lower evening cortisol ppd1-14
**Harris *et al*., 1994 [12]**	120	26.4	2w before delivery + pp	EPDS, Stein scale for maternity blues, BDI (2w before delivery), Stein (ppd1-10), psychiatric interview, Montgomery Åsberg Depression Rating Scale, EPDS (ppd35-40)		gwx morning (0800 h) + evening (2200 h); ppd1-5 morning (0800 h) + mid-day (1400 h) + evening (2200); ppd 6–40 morning (0800 h) + evening salivary cortisol (2200 h); 2w before delivery + ppd1 + 5 + 35 blood cortisol	n.s.
**Taylor *et al*., 1994 [48]**	163	N/A	ppd3	Kennerley Blues Scale, EPDS		Serum cortisol (1030 h till noon) Radioimmunoassay	Blues: higher cortisol
**Pedersen *et al*., 1993 [28]**	28	N/A	gw38 + ppw1 + 3 + 6 + 9 + 12	SCID, HRSD (gw38 + ppw1 + 3 + 6 + 9 + 12)	3 subgroups with 8, 11 resp. 8 depressed	Morning serum cortisol	DEP: higher cortisol at ppw6 than HC
**Okano and Nomura, 1992 [29]**	47	27.3	gw36 + ppd3-4 + ppm1	SADS, RDC (gw36 + ppd3-4 + ppm1)	35 HC, 12 blues	Morning serum cortisol	Blues: higher cortisol ppd3-4
**Bonnin, 1992 [30]**	29	20–39	ppw2 + 3 + 4 + 5 + 6 + 7	Zerssen self-rating scale	22 HC, 7 non pregnant HC	Morning salivary cortisol. Immunoassay	n.s. for cortisol and blues. Positive correlation between cortisol and the mood of mothers who bottle fed their babies
**O'Hara *et al*., 1991 [31]**	N/A	27 (pregnant) 27.5 (non pregnant)	gw34 + 36 + 38, ppd1 + 2 + 3 + 4 + 6 + 8	BDI, RDC, SADS (2^nd^ preg trimester + ppw9)	182 pregnant, 179 non pregnant controls	Morning blood cortisol (gw34 + 36 + 38, ppd1 + 2 + 3 + 4 + 6 + 8) and 24h urinary free cortisol (gw34 + 36 + 38, ppd 2 + 4) Radioimmunoassay; dexamethasone (ppd3)	n.s.
**Ehlert *et al*., 1990 [32]**	70	27.5	ppd1 + 2 + 3 + 4 + 5	STAI, BDI (ppd1 + 2 + 3 + 4 + 5)	29 blues	0800 h, 1300 h, 2000 h salivary cortisol Radioimmunoassay	Blues: higher cortisol during blues days and compared to HC
**Smith *et al*., 1990 [39]**	97	26.2	gw28 + gw38 + ppd2 + 3 samples during labor	POMS, MADRS (gw28 + gw38 + ppd2 + ppm3)	from gw38 to ppd2: 36 mood deterioration, 46 mood improvement	Morning blood cortisol (between 0800 h and 1000 h). Cortisol Amerlex kit	n.s.
**Harris *et al*., 1989 [45]**	147	24.6	ppw6-8	EPDS, MADRAS, Raskin scale, BDI (ppw6-8)	14.9% depressed on all three scales	Plasma and evening and morning salivary cortisol. Radioimmunoassay	n.s.
**Feksi *et al*., 1984 [33]**	40	N/A	ppd1 + 2 + 3 + 4 + 5	Symptom check list of Pitt (ppd1 + 2 + 3 + 4 + 5)	5 blues, 5 HC	Salivary cortisol 0600 h, 1200 h, 1800 h, 2200 h. Radioimmunoassay	n.s.
**Kuevi *et al*., 1983 [53]**	41	24.7	ppd2 + 3 + 4 + 5	Lubin self-rating mood scale, Luria analogue scale (ppd2 + 3 + 4 + 5)	20 no mood disturbances, 7 emotionally labile, 14 mood disturbance	Blood cortisol 1000 h + 1200 h + 2–3 h after last breastfeeding. Radioimmunoassay Amerlex cortisol kit	n.s.
**Handley *et al*., 1980 [34]**	71	26.4	gw36 + 38, ppd1 + 5	MAACL, BDI, blues index (gw36 + 38, ppd2 + 4, ppw6)	28 blues	Morning (after breakfast) plasma cortisol	Blues: higher cortisol gw38 + ppw1
**Handley et al., 1977 [55]**	18	18–31	ppd2 + 3 + 4 + 5	MAACL, BDI (ppd2 + 3 + 4 + 5)		Blood cortisol (0900–0930 h)	n.s.

ACTH: adrenocorticotropic hormone; ANX: anxiety; BDI: Beck Depression Inventory; BPRS: Brief Psychiatric Rating Scale; DEP: depressed state; DEPh: history of depression; CES-D: Center for Epidemiological Studies Depression Scale; DISC: Diagnostic Interview Schedule for Children; EPDS: Edinburgh Postnatal Depression Scale; gm: gestational month; gw: gestational week; HAM-D: Hamilton Rating Scale for Depression; HC: healthy controls; HRS-D: Hamilton Rating Scale for Depression; MAACL: Multiple Affect Adjective Check List; MADRS: Montgomery-Åsberg Depression Rating Scale; MDD: Major Depression Disorder; MINI: Mini International Neuropsychiatric Interview; N/A: not available; n.s.: non significant; PDSS: Postpartum Depression Screening Scale; PRIME-MD PHQ: Patient Health Questionnaire; PND: prenatal depression; POMS: Profile of Mood States-Depression; pp: postpartum; ppd: postpartum day; PPD: postpartum depression; ppm: postpartum month; ppw: postpartum week; preg: pregnancy; RDC: Research Diagnostic Criteria; SADS: Schedule for Affective Disorders and Schizophrenia; SCID: Scheduled Clinical Interview for DSM-IV Axis I Disorders; SCL-90-R: symptom checklist-90-R; SIGH-SAD: Structured Interview Guide for the Hamilton Depression Rating Scale, Seasonal Affective Disorders; STAI: State-Trait Anxiety Inventory; 16-PF: 16 Personality Factor Questionnaire

A variety of scales have been used to assess mood changes during the peripartum period, with the Edinburgh Postnatal Depression Scale (EPDS) [12, 19–22, 24, 27, 36, 38, 40, 43–46, 48–50, 52] and the Scheduled Clinical Interview for DSM-IV Axis I Disorders (SCID) [18, 21, 28, 37, 38, 41, 52, 54], being the most common psychometric assessment tools.

In general, findings are contradictory. While many studies show an increase in cortisol levels during pregnancy in the presence of antenatal depressive symptoms [18, 20, 24, 25, 37, 41, 46, 47, 52, 54], others have not been able to confirm such an association [12, 19, 23, 26, 38, 39, 43, 51, 52]. Likewise, a number of studies support the hypothesis of elevated peripartum cortisol levels in individuals with postpartum depression [24, 28, 40, 44] or postpartum blues [29, 32, 34, 48], while other studies point to either lower postpartum cortisol levels [27, 38, 42, 50] or no association at all [10, 12, 22, 23, 31, 39, 44, 45, 49]. These results are thus inconclusive necessitating further studies in different settings as well as in different subgroups of PPD patients. Moreover, studies focusing on evening cortisol especially are needed, as it is has only scarcely been assessed.

The present longitudinal study aimed at assessing the association between evening salivary cortisol levels and depressive symptoms during the peripartum period in a population-representative sample of women in Sweden.

## Materials and Methods

The current study was undertaken as a part of the BASIC project (Biology, Affect, Stress, Imaging, Cognition) [19, 56], a population-based, longitudinal study on psychological wellbeing during pregnancy and the postpartum period in Uppsala County, Sweden. The study was conducted at the Department of Obstetrics and Gynaecology at Uppsala University Hospital. The hospital is responsible for all delivering women within the county and for high-risk pregnancies from nearby counties. Uppsala county is a medium-sized Swedish county with a population of 346 461 inhabitants. All women attending the routine ultrasound examination are invited to participate in the study. Exclusion criteria were (1) inability to adequately communicate in Swedish, (2) protected identity, (3) pathologic pregnancies as diagnosed by routine ultrasound, (4) age less than 18 years.

The study protocol was approved by the Independent Research Ethics Board of Uppsala. All women were informed about the course and aim of the study and gave written informed consent. The investigation was carried out in accordance with the latest version of the Declaration of Helsinki.

### Study population

Between December 2011 and March 2012 as well as between June and August 2012, a total of 365 pregnant women at gestational week 36 were asked to participate in the present study. Women were instructed to complete a self-administered structured questionnaire containing a Swedish validated version of the Edinburgh Postnatal Depression Scale [57] at the 36th week of pregnancy as well as the sixth week after delivery. At both times, study subjects were also asked to collect evening cortisol salivary samples at home by using a kit sent by post along with detailed instructions.

### Psychometric measures

Depressive symptoms were assessed by use of the Swedish version of the Edinburgh Postnatal Depression Scale, an internationally-used 10-item self-reported questionnaire, designed as a screening tool to identify depressive symptoms in the peripartum period [57]. The 10 point’s threshold has often been used for biological research purposes [58, 59], as is the case in the current study, in order to increase the amount of women with possible depressive symptoms. A cut-off point of 13 for depression during pregnancy [60] and 12 for postpartum depression [61] is often used for screening in clinical settings.

The ten-item scale developed by Rosengren et al. [62] was used to assess the number of stressful life events (SLE) occurring during the past 6 months. The scale was administered via a web-based questionnaire six weeks after the delivery. An index was created, range 0–10, and in the present study, the cut-off was set at two or more SLE.

Stressful life events were included in the analyses, since they are known risk factors for PPD, but also since stress affects the HPA-axis, and therefore could possibly influence cortisol levels [63, 64].

### Sampling methods and biochemical analyses

Study subjects received written instructions to take a saliva sample between 20:00 and 22:00 hours, using Salimetrics-tubes (Electra-Box, Diagnostica AB, Sweden). This time frame was chosen to ensure maximum compliance among the participants. It should be noted that the fluctuations of cortisol levels during this interval are expected to be small [46, 65]. Participants were also instructed to refrain from any food intake, consumption of beverages, tobacco products or oral use of foreign bodies (i.e. chewing gum, toothpick or toothbrush) one hour before sampling [66]. Moreover, they were asked to report the presence of illness, oral lesions and whether they had received dental care a few days before sampling. After placing a cotton roll sublingually for at least two minutes, women stored the samples in refrigerator overnight. Thereafter, the samples were mailed back to the laboratory unfrozen, since cortisol concentrations are stable during extended periods without freezing, even when exposed to varying temperature and movement [67]. After centrifugation at 1000 *g* for 10 minutes, samples were stored in -18°C prior to further analysis. Salivary free cortisol concentrations were assessed using competitive ELISA (Salivary Cortisol Enzyme Immunoassay Kit, Salimetrics, Electra-Box, Diagnostica AB, Sweden) at the department of Laboratory Medicine at Uppsala University Hospital. The intra-assay and inter-assay coefficients of variance were 8% and 11%, respectively. All samples were run in the same assay.

Salivary free cortisol measurement was the hormone assay of choice due to its obvious practical advantages for the study subjects compared with other methods of cortisol sampling. Collection of saliva is an easy, non-invasive process that can be performed by research participants in their home environment. Moreover, salivary cortisol is a measure of the free cortisol level and cortisol is active only in the unbound state [42, 68]. This is beneficial especially in pregnant subjects as during pregnancy, altered concentrations of the corticosteroid-binding globulin may complicate the interpretation of total plasma cortisol [68]. Due to practical reasons and in order not to compromise subject representation in the sub-study, study subjects were asked to provide only one evening salivary sample before and one after delivery. This particular study focused on evening cortisol assessment, due to the scarcity of previous studies on the association between this measure and depressive symptoms during pregnancy and postpartum.

### Statistical analyses

In order to test for the normality of the cortisol levels distribution during pregnancy and postpartum, the Shapiro-Wilk test was performed and yielded a non-normal distribution. To account for non-normality, non-parametric univariate analyses were performed in order to compare cortisol levels in relation to self-reported depressive symptoms during pregnancy and postpartum as well as to various psychiatric, lifestyle, obstetric and anthropometric characteristics (Mann-Whitney *U*-test). Spearman’s rank correlation coefficient was computed to explore the correlation between peripartum cortisol levels and EPDS scores. In order to also assess differences in cortisol levels in more homogeneous groups postpartum, three groups of study participants were compared: i) healthy women, ii) women with depressive symptoms prior to or during the current pregnancy but not postpartum, and iii) women with postpartum depressive symptoms (postpartum EPDS ≥ 10), regardless of symptom onset. The Kruskal-Wallis test followed by the Dunn’s test, and/or Analysis of covariance (ANCOVA) when needing to control for confounders, were used to assess possible differences in cortisol levels in late pregnancy and postpartum, respectively, between the three groups. Cortisol levels in late pregnancy and postpartum were included in the ANCOVA models after logarithmic transformation, to account for non-normal distribution of cortisol values.

In order to assess within-subject longitudinal differences from pregnancy to postpartum, the Wilcoxon Signed Ranks Test was used. Differences between the three subgroups in the decrease in cortisol values from late pregnancy to postpartum were assessed with ANCOVA, adjusting for time elapsed from awakening to cortisol sampling and pregnancy week of sampling.

A logistic regression model was implemented, using the dichotomized postpartum EPDS score (cut-off at 10 points) as the dependent variable and cortisol levels, dichotomized at the median (comparing women with values above to those below the median) as the independent variable. In a final step, possible confounding factors were identified as those variables being associated with both cortisol levels and depressive symptoms at a significance level of *p*-value < 0.25. These were thereafter introduced in the logistic regression model. Confounding factors included poor sleep (difficulties in falling back to sleep after waking up in the night), stressful life events (0–1 vs. more events out of a ten-item scale [62]), breastfeeding (exclusive vs. non-exclusive or not at all), partner support (no or low vs. high), self-reported history of depression and use of tobacco products.

The IBM SPSS version 20 software (SPSS Inc., Chicago, IL) was used for data analysis. Statistical significance was set at a *p*-value of < 0.05.

## Results

Initially, 365 pregnant women from the BASIC cohort were invited in the current sub-study. Two hundred eighty four study subjects (78%) agreed to participate and sent in an evening salivary sample during pregnancy, and of those, 243 (67%) women also provided a postpartum salivary sample six weeks after delivery. A valid cortisol sample accompanied by completed EPDS questionnaire was available for 268 study subjects prenatally and 181 postpartum, after exclusion of outliers (cut-off standardized z-score ≥ ± 3, *N* = 5), women with a history of anorexia nervosa (*N* = 5), and invalid samples (*N* = 5, due to insufficient amount of saliva provided, as reported from the laboratory). At gestational week 36, 18.3% (49/268) of the study subjects reported significant depressive symptoms (EPDS ≥ 10). The respective prevalence at six weeks after delivery was 18.2% (33/181). The 61 women who did not return a postpartum cortisol sample were not included in further analyses. Their median cortisol was 4.0 nmol/L and their median EPDS score was 6.0 in pregnancy week 36 (data available for 58 women) and 5.0 at six weeks postpartum (data available for 45 women). Among the study subjects, eight women were treated with selective serotonin reuptake inhibitors (SSRIs) at some point during pregnancy. Their median postpartum EPDS score was 5.0 nmol/L and their median postpartum cortisol 1.14 nmol/L. These women were not excluded from analyses, as doing so did not alter the results.

Tables 2 and 3 present various anthropometric, lifestyle, obstetric, and psychiatric characteristics in relation to evening salivary cortisol levels at gestational week 36 and postpartum, respectively. In the total sample, women with self-reported postpartum depressive symptoms had significantly higher evening cortisol both in gestational week 36 (median cortisol 4.3 nmol/L vs. 3.8 nmol/L), and postpartum (median cortisol 1.19 nmol/L vs. 0.89 nmol/L) (*p* < 0.05). These associations were significant even when using an EPDS cut-off of 12 points, with median cortisol in pregnancy week 36 being 3.8 nmol/L and 4.85 nmol/L for women with low and high postpartum EPDS scores, respectively (*p* = 0.006). Likewise, the postpartum cortisol median value was 0.90 nmol/L and 1.12 nmol/L for women scoring < 12 versus ≥ 12 points on the EPDS postpartum (Mann-Whitney *U*-test, *p* = 0.025) (data not shown, available on request). However, the number of individuals classified as having depressive symptoms postpartum was then reduced to 9.6% (26/272) at gestational week 36 and 11.5% (26/227) at six weeks postpartum.

**Table 2:** Anthropometric, lifestyle, obstetric and psychiatric characteristics in relation to evening salivary cortisol levels (nmol/L) at late pregnancy stratified by self-reported depressive symptoms.

Variables		*Self-reported depressive symptoms*											
		Healthy controls			Depressive symptoms before and/or during pregnancy (not postpartum)			Depressive symptoms postpartum			Total sample		
		*N*	Median (IQR^a^)	*p* ^b^	*N*	Median (IQR^a^)	*p* ^b^	*N*	Median (IQR^a^)	*p* ^b^	*N*	Median (IQR^a^)	*p* ^b^
**Age (years)**	**20–34**	83	3.80 (1.40)	0.573	46	3.55 (1.30)	0.883	35	4.70 (2.50)	0.939	198	3.80 (1.73)	0.632
	**≥35**	35	3.80 (1.50)		17	3.50 (2.00)		8	4.20 (1.80)		71	3.90 (1.90)	
**BMI** ^c^ **(kg/m** ^**2**^ **)**	**<25**	79	3.80 (1.60)	0.799	41	3.60 (1.30)	0.762	24	4.75 (2.30)	0.990	163	3.80 (1.80)	0.941
	**≥25**	35	3.90 (1.40)		19	3.40 (1.10)		18	4.05 (1.90)		93	3.80 (1.60)	
**Education**	**Higher**	66	3.95 (1.40)	0.662	21	4.00 (2.30)	0.201	16	4.25 (1.60)	0.452	124	4.10 (1.57)	0.443
	**Lower**	14	3.90 (1.30)		10	3.25 (2.20)		8	5.10 (4.45)		35	3.80 (2.10)	
**Work**	**No**	5	3.80 (0.30)	0.372	3	3.60 (0.80)	0.590	8	4.85 (2.40)	0.344	16	3.90 (1.38)	0.833
	**Yes**	75	4.00 (1.50)		28	3.95 (2.90)		17	4.20 (2.40)		144	4.10 (1.88)	
**History ofdepression**	**No**	115	3.80 (1.50)	-	42	3.55 (2.00)	0.610	29	4.10 (1.50)	0.117	175	3.80 (1.60)	0.778
	**Yes**	0	-		21	3.50 (1.50)		14	5.35 (4.30)		69	3.80 (2.35)	
**Use of tobacco (pregnancy)**	**No**	113	3.80 (1.50)	0.119	61	3.60 (1.30)	0.350	41	4.20 (2.00)	0.159	254	3.80 (1.70)	0.728
	**Yes**	3	3.00 (0.50)		2	3.05 (0.50)		2	6.70 (3.00)		9	3.40 (3.75)	
**Stressful lifeevents** ^d^	**No**	74	3.80 (1.60)	0.469	25	3.70 (2.10)	0.233	25	4.10 (2.30)	0.503	136	4.00 (1.90)	0.369
	**Yes**	19	3.80 (1.00)		15	3.30 (1.50)		7	4.90 (4.20)		45	3.80 (2.20)	
**EPDS** ^e^ **at late pregnancy (score)**	**<10**	118	3.80 (1.50)	-	43	3.50 (1.50)	0.304	19	4.80 (2.30)	0.771	219	3.80 (1.80)	0.554
	**≥10**	0	-		20	3.85 (1.90)		23	4.30 (2.40)		49	3.90 (2.35)	
**EPDS** ^e^ **postpartum (score)**	**<10**	118	3.80 (1.50)	-	63	3.50 (1.30)	-	0	-	-	181	3.80 (1.60)	0.011*
	**≥10**	0	-		0	-		43	4.30 (2.30)		43	4.30 (2.30)	
**Week of cortisol sampling at pregnancy**	**36–37**	107	3.80 (1.50)	0.055	59	3.50 (2.10)	0.977	38	4.20 (1.90)	0.120	244	3.80 (1.70)	<0.001*
	**38–40**	11	4.40 (1.40)		4	3.50 (0.70)		5	5.80 (2.70)		29	5.10 (2.00)	
**Hours from awakening to cortisol sampling**	**≤ 12**	26	4.00 (1.80)	0.036*	26	3.50 (1.70)	0.760	20	5.10 (2.20)	0.021*	85	4.20 (2.40)	0.011*
	**≥ 13**	90	3.70 (1.20)		34	3.50 (1.90)		23	4.20 (1.70)		180	3.80 (1.70)	

^a^Interquartile range

^b^Mann-Whitney *U*-Test derived *p*-value

^c^Body Mass index

^d^Postpartum week 6, refer to past 6 months

^**e**^Edinburgh Postnatal Depression Scale

* *p* < 0.05.

**Table 3:** Anthropometric, lifestyle, obstetric and psychiatric characteristics in relation to evening salivary cortisol levels postpartum (nmol/L) stratified by self-reported depressive symptoms.

Variables		*Self-reported depressive symptoms*											
		Healthy controls			Depressive symptoms before and/or during pregnancy (not postpartum)			Depressive symptoms postpartum			Total sample		
		*N*	Median (IQR^a^)	*p* ^b^	*N*	Median (IQR^a^)	*p* ^b^	*N*	Median (IQR^a^)	*p* ^b^	*N*	Median (IQR^a^)	*p* ^b^
**Age (years)**	**20–34**	66	0.87 (0.44)	0.896	38	0.95 (0.64)	0.757	29	1.14 (0.48)	0.149	157	0.95 (0.58)	0.965
	**≥35**	30	0.90 (0.75)		14	0.95 (0.47)		4	1.43 (0.66)		57	0.91 (0.65)	
**BMI** ^c^ **(kg/m** ^**2**^ **)**	**<25**	66	0.90 (0.65)	0.186	34	0.91 (0.51)	0.696	21	1.22 (0.66)	0.956	137	0.95 (0.62)	0.552
	**≥25**	28	0.76 (0.34)		15	0.96 (0.44)		12	1.17 (0.33)		71	0.92 (0.49)	
**Education**	**Higher**	52	0.87 (0.33)	0.246	15	1.01 (0.61)	0.999	13	1.14 (0.65)	0.703	99	0.90 (0.44)	0.997
	**Lower**	9	0.76 (0.21)		8	0.89 (0.28)		4	1.04 (0.19)		24	0.89 (0.29)	
**Work**	**No**	2	0.57 (0.20)	0.079	3	0.89 (0.46)	0.573	5	1.22 (0.16)	0.387	10	0.97 (0.57)	0.847
	**Yes**	59	0.87 (0.31)		20	0.95 (0.57)		13	1.01 (0.34)		114	0.90 (0.41)	
**History of Depression**	**No**	95	0.89 (0.61)	-	34	0.89 (0.51)	0.331	24	1.22 (0.62)	0.953	146	0.92 (0.62)	0.164
	**Yes**	0	-		18	1.02 (0.55)		9	1.14 (0.20)		53	1.05 (0.43)	
**Use of tobacco (pregnancy)**	**No**	93	0.89 (0.60)	0.082	50	0.99 (0.57)	0.084	33	1.19 (0.40)	-	207	0.96 (0.57)	0.019*
	**Yes**	3	0.68 (0.34)		2	0.60 (0.08)		0	-		7	0.64 (0.19)	
**Stressful life events** ^d^	**No**	61	0.82 (0.35)	0.247	19	1.03 (0.73)	0.071	18	1.12 (0.40)	0.721	110	0.91 (0.51)	0.049*
	**Yes**	11	0.76 (0.35)		12	0.74 (0.30)		6	1.14 (0.45)		32	0.79 (0.39)	
**EPDS** ^e^ **pregnancy week 36 (score)**	**<10**	96	0.89 (0.58)	-	36	1.02 (0.59)	0.293	14	1.18 (1.06)	0.820	175	0.92 (0.61)	0.320
	**≥10**	0	-		16	0.89 (0.47)		18	1.21 (0.39)		38	1.03 (0.44)	
**EPDS** ^e^ **6 weeks postpartum (score)**	**<10**	96	0.89 (0.58)	-	52	0.95 (0.56)	-	0	-	-	148	0.89 (0.58)	0.008*
	**≥10**	0	-		0	-		33	1.19 (0.40)		33	1.19 (0.50)	
**Breastfeeding**	**No**	18	0.80 (0.64)	0.599	15	1.03 (0.66)	0.832	11	1.03 (0.22)	0.022*	55	0.92 (0.52)	0.613
	**Yes**	78	0.90 (0.44)		37	0.89 (0.48)		21	1.29 (0.55)		147	0.94 (0.61)	
**Partner support postpartum**	**No**	40	0.83 (0.53)	0.231	21	0.89 (0.55)	0.205	12	1.33 (0.61)	0.158	82	0.90 (0.59)	0.239
	**Yes**	56	0.90 (0.57)		31	1.03 (0.64)		20	1.08 (0.29)		119	1.00 (0.56)	
**Use of tobacco postpartum**	**No**	94	0.89 (0.61)	0.320	49	0.96 (0.48)	0.524	32	1.17 (0.44)	-	196	0.95 (0.57)	0.087
	**Yes**	2	0.72 (0.07)		3	0.64 (1.20)		0	-		6	0.66 (0.43)	
**Sleep problems postpartum**	**No**	41	0.94 (0.73)	0.386	21	1.10 (0.59)	0.332	15	1.06 (0.30)	0.278	75	0.89 (0.59)	0.033*
	**Yes**	55	0.87 (0.42)		31	0.89 (0.49)		17	1.29 (0.53)		121	1.03 (0.55)	
**Week of cortisol sampling postpartum**	**≤ 9**	24	0.80 (0.39)	0.299	7	1.01 (2.26)	0.647	9	1.06 (0.34)	0.490	50	0.88 (0.47)	0.719
	**≥ 10**	26	0.86 (0.26)		15	0.87 (0.44)		7	1.02 (1.18)		55	0.89 (0.33)	
**Hours from awakening to cortisol sampling**	**≤ 12**	31	0.90 (0.59)	0.544	18	0.88 (0.48)	0.349	8	1.24 (0.15)	0.486	70	1.00 (0.46)	0.985
	**≥ 13**	62	0.86 (0.56)		33	0.96 (0.59)		24	1.10 (0.65)		144	0.95 (0.63)	

^a^Interquartile range

^b^Mann-Whitney *U*-Test derived *p*-value

^c^Body Mass Index

^d^Postpartum week 6, refer to past 6 months

^e^Edinburgh Postnatal Depression Scale

* *p* < 0.05.

As demonstrated in Table 2, in late pregnancy, cortisol levels were positively associated with the pregnancy week for cortisol sampling. Moreover, there was an inverse association between late pregnancy cortisol levels and hours elapsed between awakening and cortisol sampling.

In the postpartum period (Table 3), median evening cortisol levels were significantly higher among women with sleep disturbances after delivery, whereas cortisol levels at the same point were lower among those with a history of stressful life events in the past 6 months and users of tobacco products during. Moreover, within the subgroup of study participants with postpartum depressive symptoms, median cortisol concentration was significantly higher among women who breastfed, compared to non-breastfeeding depressed study subjects. No other significant differences in cortisol levels were observed.

Finally, a significant expected decrease in cortisol values from late pregnancy to postpartum was observed, within all three subgroups (*p*-value < 0.001). However, neither the absolute nor the percentage decrease in cortisol levels was different between the three groups (after adjusting for the week of sample collection and for the elapsed time between awakening and cortisol sampling in the evening in late pregnancy).

In the postpartum period, EPDS scores were significantly and positively correlated with salivary cortisol levels (Spearman’s r = 0.166, *p* < 0.05). No significant correlation was observed between cortisol levels in pregnancy week 36 and EPDS scores in late pregnancy and postpartum.

**Fig 1:**
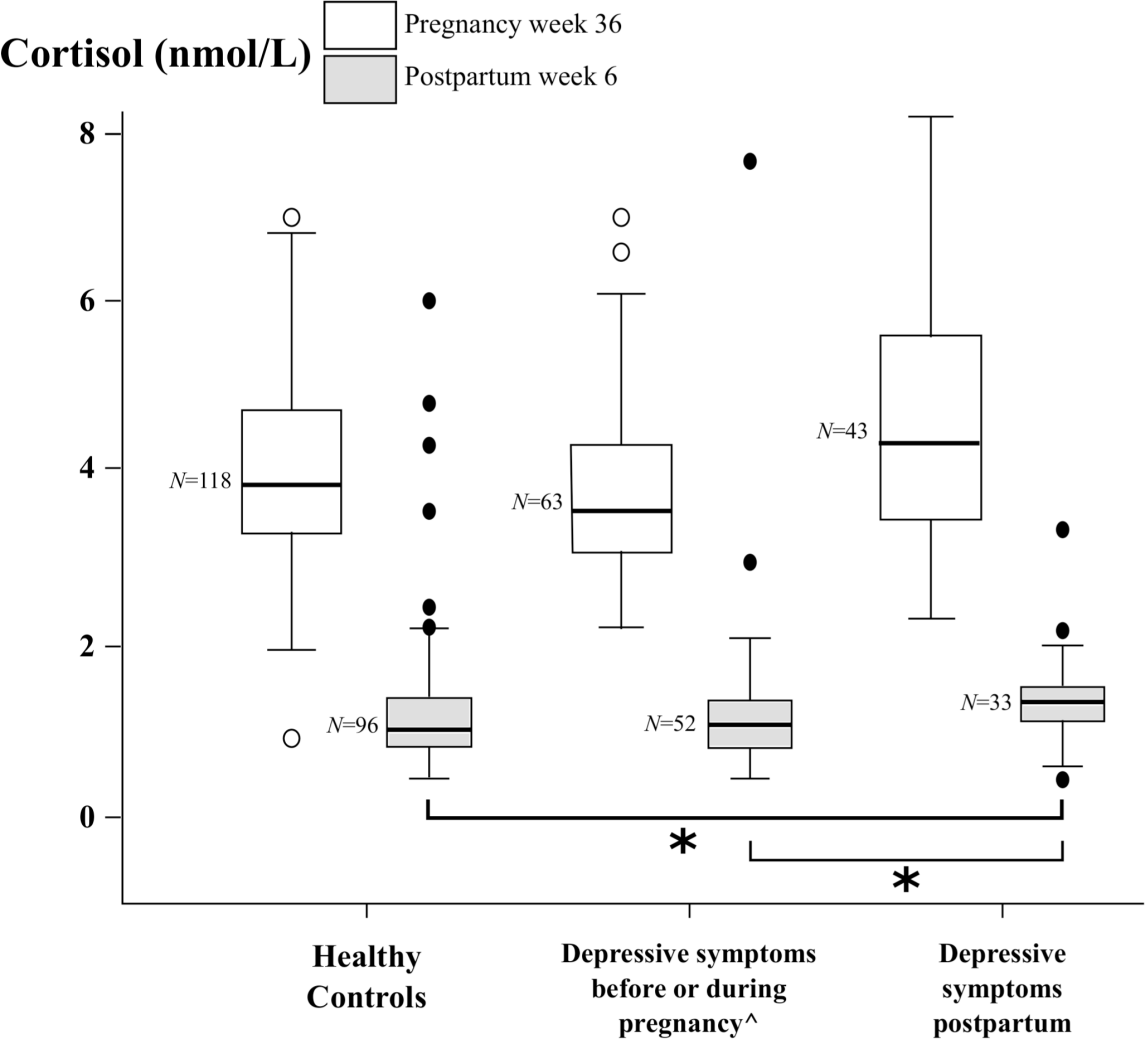
Median evening salivary cortisol values in pregnancy week 36 and postpartum week 6 among healthy controls, women with self-reported depressive symptoms before and/or during pregnancy (but not postpartum) and women with self-reported depressive symptoms postpartum. *Kruskal-Wallis test derived *p*-values (*p* < 0.05). ^but not postpartum. For illustrative purposes, cases with cortisol ˃ 8 nmol/L at pregnancy week 36 (*N* = 12) and postpartum week 6 (*N* = 5) are not shown in the figure.

As illustrated in Fig 1, study participants with postpartum depressive symptoms had significantly higher postpartum median cortisol levels compared to both women with depressive symptoms antenatally and controls (*p* = 0.019 and *p* = 0.004, respectively). In late pregnancy, a similar association between depressive symptoms and cortisol levels within the three subgroups was initially observed. However, because in late pregnancy the week of sample collection and the elapsed time between awakening and cortisol sampling in the evening had a significant association with cortisol levels in the univariate analyses, an ANCOVA model was constructed to control for these variables. After adjustment, the association between depressive symptoms and cortisol levels in late pregnancy did not remain significant.

A logistic regression model with EPDS scores postpartum as the outcome variable and a dichotomized postpartum cortisol as the exposure variable showed a positive association between cortisol levels and depressive symptoms (OR = 4.1; 95% CI 1.7–9.7). This association remained significant even after controlling for history of depression, use of tobacco, partner support, breastfeeding, stressful life events and sleep problems as possible confounders (aOR = 4.5; 95% CI 1.5–14.1).

## Discussion

The findings of the present study suggest that women with depressive symptoms in the postpartum period present with a dysregulated HPA-axis activity that is reflected in elevated postpartum evening salivary cortisol levels, compared to healthy controls. The same findings apply even when study subjects with postpartum depressive symptomatology were compared to women who experienced depressive symptoms before or during pregnancy but not after delivery.

To our knowledge, this is the first study longitudinally assessing the association between evening salivary cortisol levels in late pregnancy and postpartum with depressive symptoms at the same time-points. Several studies have attempted to elucidate the pathophysiology of peripartum depression and many suggest a central role for the HPA-axis. A summary of these studies is shown in Table 1. Although many authors have focused on cortisol secretion patterns in depressed subjects both before and after partus, methods of hormone evaluation and time point of measurement vary widely between studies and reported results are often contradictory.

Some studies, focusing on pregnancy, report higher cortisol levels in prenatally depressed women, a finding that we could not replicate. One should acknowledge that methodological differences make it difficult to directly compare study results. For example, in a multi-ethnic sample of Canadian immigrants [46], evening salivary cortisol was higher in pregnant subjects with depression compared to healthy controls. However, cortisol was assessed in pregnancy week 19 and the sample was not population representative. Similarly, a number of studies describe that dysthymia in week 18 [54] or depression in midgestation [18, 25] may be related to increased urinary cortisol. Voegtline et al. [47] illustrate higher mid-afternoon salivary cortisol in depressed pregnant subjects in week 30–32 while Meliska et al. [52] report an association between elevated mesor cortisol (mesor cortisol being an estimate of the circadian rhythm-adjusted mean cortisol level), though not evening cortisol, and high depression scores up to pregnancy week 34. O’Keane et al. describe a positive association between prenatal depressive symptoms and evening salivary cortisol in pregnancy week 32, in a small sample of women. However, the authors do not mention if the exact pregnancy week or the time point of sampling during the evening were taken into account [37].

In addition, the large inter-individual variation that characterizes cortisol levels during pregnancy, and the fact that a single evening measurement may not fully depict the dynamic cortisol secretion in pregnant women [19], may also have contributed to the non-significant association between cortisol levels and depression during late pregnancy in the present findings.

Other studies have attempted to establish an association between high postpartum cortisol levels and postpartum blues. The term postpartum blues refers to a transient change in mood occurring in the first days after delivery and has been associated with development of full-blown postpartum depression [48, 69]. A study by Taylor et al. found a positive association between morning serum cortisol levels and postpartum blues [48]. Similarly, another study [29] showed that women with postpartum blues had higher morning serum cortisol at the third and fourth day postpartum, compared to healthy controls. In another study, where salivary cortisol was measured among women in the early postpartum period, the authors suggested morning salivary cortisol as a possible marker for postpartum blues [32].

Among studies focusing on postpartum mood, Taylor et al. report significantly elevated levels of morning salivary cortisol among women with postpartum depressive symptoms, compared to healthy controls. These results point to an abnormal function of the HPA-axis that is also associated with a reduced morning rise in the 30 minutes post-awaking cortisol levels in depressed women [40]. A similar pattern of cortisol elevation in postpartum depression is also supported by another study where subjects with high EPDS scores postpartum presented with high afternoon serum cortisol levels [24]. In line with findings from studies in non-pregnant depressed subjects [70], it can be hypothesized that the prolonged elevated secretion of corticotropin releasing hormone in the locus coeruleus might contribute to the higher cortisol levels observed in depressed women during the postpartum period.

Similarly, Pedersen et al. suggest that high levels of puerperal cortisol in depressed women postpartum may be associated with a diminished negative feedback and/or increased central drive within the HPA-axis. The authors found that depressed women at six weeks postpartum had significantly higher levels of morning serum cortisol [28]. Although the studies named above are not methodologically identical to our study, they all point to a state of an HPA-axis alteration and possibly HPA-axis hyper regulation that may increase the risk for depressive symptoms postpartum. Moreover, our main results seem to be supported by some similar studies in non-pregnant subjects, which demonstrate that morning salivary cortisol may constitute a biomarker for depressive episodes occurring within the first years following cortisol assessment [71–74].

On the other hand, a number of studies that attempted to evaluate HPA dysregulation and PPD point to lower cortisol levels among depressed women postpartum. Possible explanations for the contradictory results might include methodological differences concerning the timing of the hormone sampling during the day, the hormone method used for assessing cortisol, i.e. blood, urine or salivary sampling, as well as PPD cases of different severity or subtype, that might be more prominent in some settings. For example, Saleh et al. [50], found a higher drop in cortisol between pregnancy and postpartum among depressed subjects as well as lower mean postpartum cortisol levels compared to healthy controls, when analysing morning blood cortisol samples one week postpartum. In another study [27], the authors report an association between lower evening salivary cortisol and PPD. However, these findings are difficult to directly be compared to the present results since cortisol samples were taken at a different time-point (from 14 days before up to ten days after delivery) than the mood assessment (six weeks postpartum). Furthermore, Groer and Morgan [42] found a negative association between postpartum depression and salivary cortisol. As the authors suggest, the pregnancy hypercortisolism may affect the HPA-axis regulation in the postpartum period with decreased feedback sensitivity to cortisol at the hypothalamus and pituitary. It should be however noted that in this study, salivary cortisol was assessed in morning samples. Lastly, a number of studies assessing cortisol levels in relation to prenatal and postpartum depressive symptoms, presented in Table 1, yielded non-significant results [19, 23, 36, 43, 49, 51]. The large variations in terms of sample size, subgroups, hormonal and psychometric measures among these studies make the interpretation of the results and the drawing of conclusions rather difficult.

It has previously been suggested that depression during pregnancy and postpartum may have different pathophysiological mechanisms. In this theoretical model, depression during pregnancy resembles melancholic depression characterized by HPA-axis hypersensitivity and high levels of cortisol. During the postpartum period, the normally elevated CRH occurring during late pregnancy, may down-regulate hypothalamic CRH and cortisol output and increase the risk for depression [16]. This pathophysiological mechanism has also been proposed in patients with atypical depression [17, 75]. Furthermore, Magiakou et al. [10] described a prolonged blunting of the HPA-axis in women with postpartum depression. On the contrary, Bloch et al. [76] created an endocrine model of pregnancy and the postpartum period using gonadal hormonal add-back to mimic the hormonal fluctuations during the normal pregnancy and puerperium by stimulating the HPA-axis with ovine CRH. This study showed a hyperregulation of the HPA-axis in the presence of high concentrations of gonadal hormones, more profound among PPD subjects, whereas no differential effect was found during the subsequent withdrawal phase (equivalent to the postpartum period).

In the current study, there was no significant difference in the absolute or relative decrease of cortisol levels, from late pregnancy to after delivery, between the three subgroups (healthy, depressed during pregnancy and depressed postpartum). It is possible that elevated postpartum cortisol levels *per se*, and not the alteration in cortisol levels subsequent to the delivery of placenta, contribute to the occurrence of postpartum depressive symptoms. In fact, this pathophysiological pattern has been described in major depression among non-pregnant subjects, since a number of studies suggest a positive association between cortisol levels and major depression [77, 78].

The results of the current study replicate previous findings on associations between cortisol levels and postpartum variables, such as sleep problems [79] and stressful life events. Study subjects who experienced two or more stressful life events during the past six months had lower evening salivary cortisol at six weeks postpartum compared to controls. A number of other studies have found a negative association between salivary cortisol and stressful life events during pregnancy [36, 43, 64], indicating a dysregulated HPA-axis. On the contrary, in a study by Obel et al. [80], women having experienced stressful life events during pregnancy tended to have higher evening cortisol levels during late pregnancy. Additionally, a similar pattern has been reported in non-pregnant subjects with posttraumatic stress disorder [81]. These seemingly contradicting results may suggest a differential effect of stress on cortisol levels and HPA-axis in general, with regard to timing and duration of exposure. Moreover, a positive association between evening cortisol levels and lactation was observed in women with significant postpartum depressive symptoms. A recent study reported higher levels of cortisol in breastfeeding symptomatic women, compared to asymptomatic [82]. On the other hand, previous studies have suggested a protective effect of breastfeeding on postpartum depression, through attenuation of cortisol stress response [83–85]. However, these studies were not conducted in depressed women and the hormonal sampling method and time-point differed from the ones in the current study. Thus, the present finding needs to be interpreted with caution, especially since it was not observed an effect of breastfeeding in the total sample, but only among depressed subjects. Additionally, the inclusion of breastfeeding as a possible confounder in the logistic regression model did not influence our results.

In the postpartum period, a positive, yet modest, correlation between cortisol levels and depressive symptoms was observed in the total study population. It should be noted that the results of the present study, while contributing to our understanding of the role of HPA-axis in peripartum depression, do not suggest that evening salivary cortisol alone could be used for the prediction of women at risk. It should also be noted that this study identified differences in evening salivary cortisol levels between distinct subgroups of women in the peripartum period. Further studies on the role of HPA-axis in peripartum depression might also consider analysing separately subgroups of perinatally depressed women.

Among the strengths of this study are its population-based, longitudinal design, and the availability of information on a large number of possible confounding factors. Moreover, the assessment of cortisol levels in saliva contributes to a more accurate evaluation of the hormonal status of study subjects, as this method is a reliable measure of free cortisol levels. A study limitation is the use of a self-reporting psychometric measure instead of a psychiatric interview which may have been more accurate but also more difficult to conduct in a research setting. The EPDS is a self-reporting instrument, and thus a degree of misclassification may occur. However, this scale is widely used and validated and has a quite high sensitivity and specificity [61, 86, 87]. Another limitation consists in the use of a self-sampling kit for salivary cortisol sampling, which was performed at the home environment, making it difficult to control for inconsistencies in the sampling procedure. The provision of multiple cortisol samples has been advocated [88], since a series of measurements would more precisely depict the HPA-axis activity [89]. However, because it would severely compromise participation rates, single sampling during late pregnancy and postpartum was finally chosen to be included in the protocol of this study. It is of note that possible misclassification of cortisol values because of single sampling would affect participants in all three subgroups, and would be expected to have attenuated any existing associations. Finally, data on physical activity were not collected at the six-week postpartum time-point, when many women may change their habits.

## Conclusions

The findings of the present study suggest that women with depressive symptoms in the postpartum period present with a dysregulation in HPA-axis activity that is reflected in elevated evening salivary cortisol levels postpartum, considering confounding factors.

## Supporting Information

S1 Dataset All data used for the statistical analyses has been made available to the publishing journal. (XLSX)
